# Comparison of RCAS1 and metallothionein expression and the presence and activity of immune cells in human ovarian and abdominal wall endometriomas

**DOI:** 10.1186/1477-7827-4-41

**Published:** 2006-08-14

**Authors:** Lukasz Wicherek, Magdalena Dutsch-Wicherek, Krystyna Galazka, Tomasz Banas, Tadeusz Popiela, Agata Lazar, Beata Kleinrok-Podsiadlo

**Affiliations:** 1 Department of Gynecology and Infertility of the Jagiellonian University, 23 Kopernik Str, 31-501 Krakow, Poland; 2 Department of Pathomorphology of the Jagiellonian University, 17 Grzegorzecka Str, 31-531 Krakow, Poland; 3 ENT Department of the Jagiellonian University, 2 Sniadeckich Str, 31-531 Krakow, Poland; 4 Department of the General Surgery of the Jagiellonian University, 40 Kopernik Str, 31-501 Krakow, Poland

## Abstract

**Background:**

The coexistence of endometrial and immune cells during decidualization is preserved by the ability of endometrial cells to regulate the cytotoxic immune activity and their capability to be resistant to immune-mediated apoptosis. These phenomena enable the survival of endometrial ectopic cells. RCAS1 is responsible for regulation of cytotoxic activity. Metallothionein expression seems to protect endometrial cells against apoptosis. The aim of the present study was to evaluate RCAS1 and metallothionein expression in human ovarian and scar endometriomas in relation to the presence of immune cells and their activity.

**Methods:**

Metallothionein, RCAS1, CD25, CD69, CD56, CD16, CD68 antigen expression was assessed by immunohistochemistry in ovarian and scar endometriomas tissue samples which were obtained from 33 patients. The secretory endometrium was used as a control group (15 patients).

**Results:**

The lowest metallothionein expression was revealed in ovarian endometriomas in comparison to scar endometriomas and to the control group. RCAS1 expression was at the highest level in the secretory endometrium and it was at comparable levels in ovarian and scar endometriomas. Similarly, the number of CD56-positive cells was lower in scar and ovarian endometriomas than in the secretory endometrium. The highest number of macrophages was found in ovarian endometriomas. RCAS1-positive macrophages were observed only in ovarian endometriomas. CD25 and CD69 antigen expression was higher in scar and ovarian endometriomas than in the control group.

**Conclusion:**

The expression of RCAS1 and metallothionein by endometrial cells may favor the persistence of these cells in ectopic localization both in scar following cesarean section and in ovarian endometriosis.

## Background

The ovary is the most common location of the ectopic endometrium occurrence in the pelvic genital organs. Endometriosis is also found outside the genital tract. The cesarean scar was the most common site of extragenital endometriosis [1]. It was suggested in the 1950s that endometrioma occurring in a cesarean scar might result from specific endometrial changes depending on the pregnancy development [2]. Ovarian endometriosis, however, was thought to be associated with retrograde menstruation [3]. The aforementioned two hypotheses in combination with the current opinion indicating that the endometrial tissue acquires a secondary gestagen resistance [4] indicates the natural ability of endometrial cells to coexist with adjacent activated immune cytotoxic cells within the decidua. This phenomenon is secondary to the participation of endometrial cells in reproduction and enables the creation of maternal immune tolerance against fetal antigens.

The cells of the ectopic endometrium preserve an ability of eutopic endometrial cells to regulate the cytotoxic immune activity, for example, by the expression of many immunomodulating factors (IL-1, IL-6, IL-8 and others) [5] and by their resistance to immune-mediated apoptosis. A lower sensitivity to immune-induced apoptosis was noticed in endometrial cells in ovarian cysts [6]. Both features seem to be crucial for endometrial cell survival in ectopic localization.

RCAS1, which until now has been thought to be responsible for tumor escape from immune surveillance in various human cancer cells [7-9] seems to be an important factor for cytotoxic activity regulation in the endometrium [10]. The expression of RCAS1 has been detected in the bone marrow, the Waldeyer's ring, the placenta, the endometrium, and the tubal mucosa thus indicating its role in immune cells regulation [11-13].

Metallothionein (MT) is a cysteine-rich, low molecular weight cytoplasmic protein. Its expression is related with both processes concerning cell proliferation and cell death [14]. MT expression correlated in cancer tissue with reduced apoptosis in carcinoma cells [15]. Its expression was observed in the endometrium and it was significantly higher in the secretory than in the proliferative phase with a peak during the implantation window [10,16,17]. MT expression was also demonstrated in endometrial cancer and ovarian endometriosis [16]. MT expression seems to protect endometrial cells against apoptosis enabling them to acquire resistance to immune-mediated apoptosis [10].

The absence of pelvic endometriosis is typical in patients with abdominal wall endometrioma [18]. The reported incidence of cesarean scar endometriosis ranges between 0.03 and 0.047 per cent and is reported until 2 years after the surgical procedure [19]. The pelvic ectopic endometrium might undergo decidualization during pregnancy even in ovarian endometriosis, where the initiation of implantation starts in the early proliferative phase. On the contrary, scar endometriosis may form chocolate cysts although it starts from the implantation of a decidual cell. The formation of the ectopic decidua in ovarian endometrioma is a well documented phenomenon. Deciduosis is usually an asymptomatic phenomenon and continues undetected throughout pregnancy [20]. The ectopic endometrium preserves the ability to undergo reversible decidualization, which is a phenomenon typifying normal eutopic endometrium.

The aim of the present study was to evaluate RCAS1 and metallothionein expression in ovarian and scar endometriomas in relation to immune cell presence and their activity.

## Materials and methods

### Subjects

Forty-eight patients were included in our study. The material was collected during routine surgical procedure in the Department of Gynecology and Infertility of the Jagiellonian University in Krakow, Poland between January and November 2005. No patient included in our study received any hormonal treatment. Patients' consent was obtained in all cases. The approval of the research program by the Jagiellonian University Ethical Committee was obtained prior to the study (KBET/89/B/2005). The tissue samples were obtained during routine surgical procedures, were immediately fixed in 10% buffered formaldehyde solution and sent to the Pathomorphology Department of the Jagiellonian University. Two experienced pathomorphologists (K.G. and A.L) independently evaluated the routinely stained (hematoxylin and eosin) slides prepared from paraffin-embedded tissue material, and selected the material adequate for further analysis. Paraffin blocks were cut and used for immunohistochemistry.

### Ovarian endometriomas

Ectopic human endometrium tissues were obtained from 19 non-menopausal infertile women, aged 25–37 years from ovarian lesions during laparoscopic cyst enucleation. Before surgery the patients complained of dysmenorrhoea (12 cases), dyspareunia (14 cases) and chronic pelvic pain (10 cases).

### Abdominal wall endometriomas

Scar endometriomas were diagnosed in 15 patients aged 25–35 years in the incisional scar after cesarean elective delivery with the presence of subcutaneous nodules infiltrating the fascia (10 cases) and muscle (5 cases). The surgical procedure was performed less than two years after cesarean section. The patients complained of a cyclic local pain and tenderness at the time of menstruation. Patients after cesarean section with multiple pregnancies or existing pregnancy complications such as preterm deliveries, hypertension, diabetes mellitus, and cases of fetal demise were excluded from this study.

### Control group

Eutopic human endometrium tissues were obtained from 14 non-menopausal fertile women, aged 29–41 years. These patients underwent hysterectomy because of a benign gynecological indication (leiomyomas). Tissue samples were classified according to the menstrual cycle phases. We included into the study only the tissue samples from the mid secretory cycle phase. The control samples from uterine corpus included the entire thickness of the endometrium (basal and superficial part, composed of stromal cells and glandular epithelial cells).

### Immunohistochemistry

Immunohistochemical analysis was performed in the Pathology Department of the Jagiellonian University. Five-micrometer sections from each case were stained to visualize expression of RCAS1, MT and CD16-, CD25-, CD69-, CD56-positive cells (mainly lymphocytes) as well as CD68+ cells, or macrophages. In all cases immunohistochemistry was performed applying Envision method using Dako Autostainer. The following antibodies were applied: mouse monoclonal antibody Anti-RCAS1 (Medical and Biological Laboratories, Naka-ku Nagoya, Japan in DAKO Antibody Diluent with Background Reducing Components-DAKO, Denmark, dilution 1:1000), monoclonal mouse antibody ImmunOTM (MP Biomedicals, Inc., clone 1A12 in dilution 1:1000), CD56 (NCAM; NCL-CD56-504, Novocastra) in dilution 1:100, CD69 (NCL-CD69, Novocastra) in dilution 1:25, CD25 (Interleukin-2 Receptor, NCL-CD25-305, Novocastra) in dilution 1:25, CD16 (NCL-CD16, Novocastra) in dilution 1:40, CD68 (Klone PG-M, Dako) in dilution 1:50, according the manufacturer's instructions. Visualization of reaction products was performed using AEC (3-amino-9-ethyl-carbazole) as a chromogen (AEC Substrate Chromogen ready-to-use, DAKO, Denmark) for 10 minutes at room temperature. Sections were counterstained with hematoxylin and mounted in glycergel. As a positive control a tonsil specimen was taken for RCAS1 and a breast cancer specimen for metallothionein. All stainings were performed with the same procedure but with the omission of the primary antibody as a negative control. RCAS1 expression was evaluated in entire slides in endometriosis area, in glandular epithelium (superficial and of the glands) and the stromal cells (fibroblasts), considering the per cent of positive cells and the intensity of the colour reaction. The degree of metallothionein positivity was quantified as the percentage of MT-positive cells in the endometriosis lesion. The staining in epithelial and stromal cells of endometriosis was evaluated. The scales used for estimation of both marker staining are shown in Table 1.

**Table 1:** The scale used for evaluation of metallothionein and RCAS1 expression.

Antigen	Immunoreactivity			
	
	0	+1	+2	+3
RCAS1	No reactivity	Weak (when observed any, also granular in paranuclear region) cytoplasmic staining pattern in up to 10% of positive cells	Marked cytoplasmic (sometimes together with membranous) staining in 11–30% of the cells	High expression – more than 30% of positive cells
Metallothionein	Lack of any positivity	Weak staining in less than 5% of the cells	Moderate – various staining intensity but in <50% of the cells,	Strong – staining of more than 50% of the cells.

The immune cells were calculated in an entire specimen, in the region of endometriosis and an average cell number per 1 hpf (high power field, objective magnification ×40) was calculated. Variable scales were used to evaluate semiquantitatively an amount of the cells, depending on their general number in the specimen, summarized in Table 2.

**Table 2:** The scale used for evaluation of CD25, CD56, CD68, CD69, and CD16 antigens expression.

Antigen	Immunoreactivity				
	
	0	1+	2+	3+	4+
CD25	Lack of positive cells	Single positive cells	1–5 positive cells/1 hpf	More than 5 positive cells/1 hpf	-
CD56	Lack of positive cells	Single positive cells	1–5 positive cells/1 hpf	More than 5 positive cells/1 hpf	-
CD69	Lack of positive cells	Single positive cells	1–5 positive cells/1 hpf	More than 5 positive cells/1 hpf	-
CD68	Lack of positive cells	1–5 positive cells/1 hpf	6–10 cells/1 hpf	11–20 positive cells/1 hpf	More than 20 positive cells/1 hpf
CD16	Lack of positive cells	1–5 positive cells per 1 hpf	6–10 cells/1 hpf	11–20 positive cells/1 hpf	More than 20 positive cells/1 hpf

The evaluation of immunohistochemical reactions was performed independently by two histopathologists (K.G. and A.L.).

### Statistical analysis

The distribution of variables in the study groups of women checked with the use of the Shapiro-Wilk test showed that all of them were different from normal. Therefore, nonparametric testing was employed. Statistical significance between the groups was determined by the Kruskal-Wallis analysis of variance (ANOVA) test. The Mann-Whitney U test was then used as applicable. The Spearman rank test was used to evaluate interclass correlation coefficients. All calculations were carried out with the use of STATISTICA software v. 6 (StatSoft, USA, 2001).

## Results

RCAS1 immunoreactivity was visible in the endometrial epithelium without positive reaction in the proper stromal cells.

**Figure 1:**
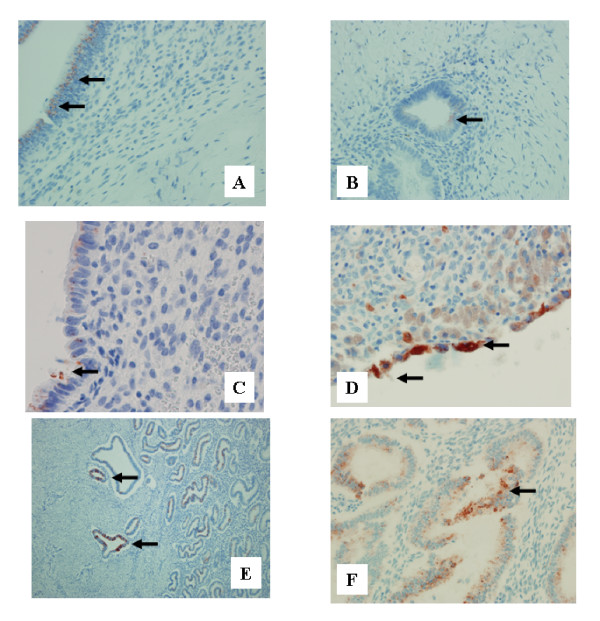
RCAS1 expression in: scar endometriomas (A,B), ovarian endometriomas (C,D) and secretory endometrium (E,F). A – Weak positive glandular reaction in scar endometrioma (horizontal arrows). Obj. magn. ×40; B-Very weak immunoreactivity in glandular epithelium in scar endometrioma (horizontal arrow). Obj. magn. ×40; C – Weak immunoreactivity in glandular epithelium of ovarian endometrioma (horizontal arrow). Obj. magn. ×60; D – Strong immunoreactivity in glandular epithelium of ovarian endometrioma (horizontal arrows). Obj. magn. ×60; E – Strong immunoreactivity in glandular epithelium of the secretory endometrium, also of its basal part (horizontal arrows). Obj. magn. ×10; F – Strong immunoreactivity in glandular epithelium of secretory endometrium (horizontal arrow). Obj. magn. ×40.

**Figure 2:**
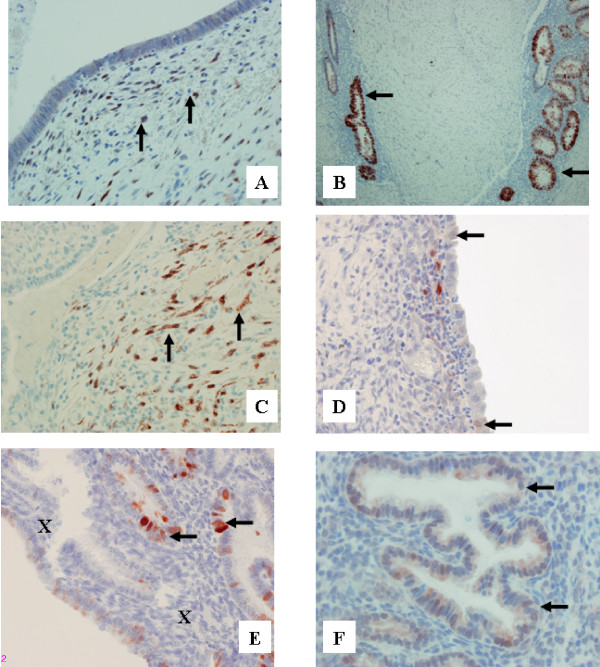
MT expression in: scar endometriomas (A,B), ovarian endometriomas (C,D) and secretory endmetrium (E,F). A – Moderate positive stromal reaction in scar endometrioma tissue (vertical arrows). Obj. magn. ×40; B-Strong immunoreactivity in glandular epithelium of scar endometrioma (horizontal arrow) Obj. magn. ×10; C – Moderate immunoreacitvity in stromal cells of ovarian endometrioma (vertical arrows). Obj. magn. ×40; D – Very weak immunoreactivity in glandular epithelium of ovarian endometrioma (vertical arrows). Obj. magn. ×40; E – Strong immunoreactivity in glandular epithelium of secretory endometrium (horizontal arrows) and negative stromal cells (x). Obj. magn. ×40. F – Strong MT immunoreactivity in glandular epithelium of the secretory endometrium (horizontal arrow). Obj. magn. ×60.

RCAS1 immunopositivity was revealed in 57% of ovarian endometriosis tissue samples, in 60% of scar endometrioma tissue samples and in all examined secretory endometrial tissue samples (Figure 1.) The differences in the distribution of MT immunoreactivity were found between stromal cells and glandular cells in the tissue samples derived from the scar and ovarian endometriosis. No such alterations in MT distribution were observed between samples in the control group (Figure 2.) Metallothionein immunoreactivity was observed in glandular epithelium of 21% ovarian endometriosis tissue samples, 94% scar endometriomas and in all secretory endometrium tissue samples. Metallothionein immunopositivity was shown in stromal cells of 69% ovarian endometriosis tissue samples and in all stromal cells of scar endometriomas. No MT immunoreactivity was observed in stroma of secretory endometrium. Immune cell presence (CD68, CD56 and CD16) and activity (CD69, CD25) were assessed within stroma.

The statistical analysis of examined factors in ovarian endometriosis, scar endometriomas and secretory endometrium was performed by the Kruskal-Wallis analysis of variance (ANOVA) test and the results are presented in Table 3.

**Table 3:** The results of statistical analysis of metallothionein (MT), RCAS1, CD68, CD56, CD16, CD25 and CD69 expression in ovarian endometriosis, scar endometriomas and secretory endometrium performed by Kruskal-Wallis analysis of variance (ANOVA) test. (± SE – standard error).

Antigens	Ovarian endometriosis	Scar endometriosis	Control group	p
RCAS1	0.684 (± 0.149)	0.666 (± 0.168)	1.583 (± 0.173)	0.0026
MT – glandular epithelium	0.211 (± 0.159)	1.800 (± 0.179)	2.167 (± 0.185)	<0.001
MT – stroma	1.000 (± 0.162)	2.200 (± 0.182)	0.000 (± 0.189)	<0.001
CD56	0.579 (± 0.135)	0.400 (± 0.152)	2.000 (± 0.186)	<0.001
CD16	1.789 (± 0.240)	1.600 (± 0.270)	2.300 (± 0.331)	0.292
CD68	2.579 (± 0.227)	2.000 (± 0.256)	2.800 (± 0.313)	0.024
CD25	0.421 (± 0.239)	1.667 (± 0.268)	0.300 (± 0.329)	0.009
CD69	0.895 (± 0.259)	1.200 (± 0.292)	0.300 (± 0.357)	0.167

### Analysis of RCAS1 immunoreactivity

**Figure 3:**
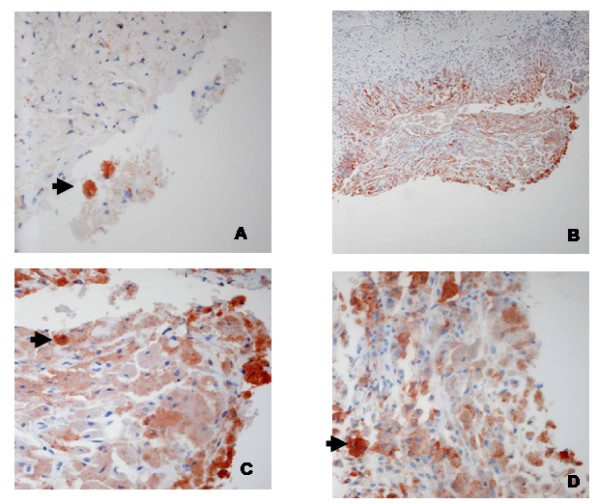
Destroyed epithelial layer of an endometrial ovarian cyst. The covering of it composed mainly of macrophages, with strong RCAS1 expression (B, C, D). RCAS1-positive macrophages present also loosely in the cyst fluid (A). Obj magn.: A, C, D ×40; B ×20.

Statistically significant lower RCAS1 expression was observed in ovarian endometriosis than in the control group (p = 0.002). Similarly, statistically significant lower RCAS1 expression was identified in scar endometriosis in comparison to the control group (p = 0.002). RCAS1 expression was on comparable levels in scar and ovarian endometriosis (Table 4.) The RCAS1 positive macrophages were found in ovarian endometriomas, but they were not detected either in the eutopic endometrium or in scar endometriomas (Figure 3.)

**Table 4:** The immunhistochemical analysis of RCAS1 expression in glandular epithelium

Groups	Part of endometrium	RCAS1 immunoreactivity*			
		
		0	+1	+2	+3
Ovarian endometriosis (n = 19)	Glandular epithelium	42 (8)	47 (9)	11 (2)	-
Scar endometriosis (n = 15)	Glandular epithelium	40 (6)	53 (8)	7 (1)	-
Control group (n = 14)	Glandular epithelium	-	57 (8)	35 (5)	8 (1)

*percentage of cases; (n-total number of cases)

### Analysis of MT immunoreactivity

A statistically significant lower MT immunoreactivity in the glandular epithelium was identified in the ovarian endometriosis in comparison to the scar endometriosis (p < 0.001) and was also statistically significantly lower than in the control group (p < 0.001). No differences in MT expression in the glandular epithelium were observed between scar endometriosis and the control group (Table 5.) MT immunoreactivity was statistically significantly higher in the stroma of scar endometriosis when compared to ovarian endometriosis (p < 0.001).

**Table 5:** The immunhistochemical analysis of metallothionein expression in the glandular epithelium and stroma.

Groups	Part of endometrium	Metallothionein immunoreactivity*			
		0	+1	+2	+3
Ovarian endometriosis (n = 19)	Glandular epithelium	78 (15)	22 (4)	-	-
	Stroma	31 (6)	38 (7)	31 (6)	-
Scar endometriosis (n = 15)	Glandular epithelium	7 (1)	27 (4)	46 (7)	20 (3)
	Stroma	-	27 (4)	27 (4)	46 (7)
Control group (n = 14)	Glandular epithelium	-	28 (4)	44 (6)	28 (4)
	Stroma	100(14)	-	-	-

*percentage of cases; (n-total number of cases)

The difference in MT expression between the ovarian glandular epithelium and ovarian stroma was observed and was statistically significant (p = 0.01). However, no differences of MT expression in scar endometriosis between the glandular epithelium and the stroma were found.

### Analysis of immune cells presence (Table 6)

**Table 6:** Density of CD56+, CD16+, CD68+, CD25+ and CD69+ cells in the endometrial stroma.

Tissues samples	CD – antigens	The number of lymphocytes				
		0	+1	+2	+3	+4
Ovarian endometriosis (n = 19)	CD56	47 (9)	47 (9)	6 (1)	-	-
	CD16	6 (1)	41 (8)	26 (5)	21 (4)	6 (1)
	CD68	-	22 (4)	26 (5)	26 (5)	26 (5)
	CD25	78 (15)	22 (4)	-	-	-
	CD69	47 (9)	26 (5)	15 (3)	12 (2)	-
Scar endometriosis (n = 15)	CD56	66 (10)	27 (4)	7 (1)	-	-
	CD16	19 (3)	27 (4)	27 (4)	27 (4)	-
	CD68	11 (2)	11 (2)	37 (5)	41 (6)	-
	CD25	41 (6)	-	11 (2)	48 (7)	-
	CD69	48 (7)	19 (3)	7 (1)	19 (3)	7 (1)
Control group (n = 10)	CD56	-	10 (1)	80 (8)	10 (1)	-
	CD16	-	20 (2)	40 (4)	30 (3)	10 (1)
	CD68	-	20 (2)	80 (8)	-	-
	CD25	70 (7)	30 (3)	-	-	-
	CD69	80 (8)	10 (1)	10 (1)	-	-

*percentage of cases; (n-total number of cases)

The number of macrophages was significantly higher in ovarian endometriosis in comparison to the control group, similarly it was observed to be statistically significantly higher in scar endometriosis than in the control group (p = 0.03).

CD69 antigen expression was on comparable levels in ovarian and scar endometriosis, while in ovarian endometriosis it was significantly higher than in the control group. Similarly, CD69 antigen expression was significantly higher in scar endometriosis than in the control group. CD25 antigen expression was statistically significantly higher in scar endometriosis than in the control group (p = 0.02). No differences in CD25 expression were identified between ovarian endometriosis and the control group. Statistically significantly lower CD25 expression was detected in ovarian than in scar endometriosis (p = 0.005). The number of CD56-positive cells was identified in ovarian and scar endometriosis on comparable levels, but it was in both cases statistically significantly lower than in the control group (p < 0.001). No differences in CD16 antigen expression were observed between the studied tissue samples and the control group.

## Discussion

RCAS1 immunoreactivity was more prominent in the secretory endometrium than in scar and ovarian endometriomas. Stronger Metallothionein expression was determined in scar endometriomas and in secretory endometrium than in ovarian endometriosis. The alterations in RCAS1 and Metallothionein expression are accompanied by changes in CD25, CD56 and CD69 antigens expression on immune cells.

A decrease of CD25 antigen expression with a concomitant increase of CD69 expression has been observed in the endometrium according to the menstrual cycle changes [21]. Chao et al. concluded that there might exist a factor or factors capable of selective suppression of activated cytotoxic cells. RCAS1 could be considered as a factor to play that role. Its expression increases significantly during the secretory cycle phase. In our previous report a significantly lower RCAS1 expression was noticed in ovarian endometriosis in comparison to endometrial carcinoma [10]. RCAS1 expression was growing in the eutopic endometrium simultaneously with the growth of the number of CD56-positive cells and CD69 antigen expression. In the present study a correlation between CD56 and CD69 expression was observed in ovarian endometriomas (R = 0.53, p = 0.018) as well as in scar endometriomas (R = 0.52, p = 0.04). A decrease of the number of CD56-positive cells in scar and ovarian endometrioses seems to remain in agreement with described NK cell dysfunction, which is considered to contribute to the pathogenesis of endometriosis [22]. Diminished NK cell activity has been observed in the peritoneal fluid of women with endometriosis [23]. The main task of scientists is focused on the question whether this dysfunction is primary or secondary to the development of ovarian endometriosis. The expression of the killer inhibitory receptors (KIRs; for example CD158a) on NK cells associated with the decrease of the NK cell activity might indicate that the NK cell changes are the primary event [22]. On the contrary, a higher expression of class I human lymphocyte antigens on endometrial cells surviving in ectopic localization [24] suggests the secondary resistance to natural killer-mediated cytolysis [25]. The RCAS1 expression observed in this study in both scar and ovarian endometriomas points to the possibility of inhibition of cytotoxic immune response, which might be secondary to the physiological ability of endometrial cells to regulate immune cell activity. The decreased RCAS1 expression in scar and ovarian endometriomas in comparison to the eutopic secretory endometrium might result from the lower number of NK cells. NK dysfunction in endometriosis was found to be related to the diminished macrophages ability to present antigens [26].

Macrophages seem to play a central role in the immunobiology of endometriosis [27]. The recent evidence suggests that ovarian macrophages migration is a result of RANTES secretion into the environment of endometriomas [28], and their numerous products (IL-1beta, TNF-alfa, Il-6, Il-8, MMP-9, VEGF and others) may be involved in the onset and development of endometriosis [27]. In our present study the highest number of macrophages was identified in ovarian endometriosis when compared to scar endometriosis and to the control group. Interestingly, only ovarian macrophages were RCAS1 positive (Fig. 3). The presence of RCAS1-positive macrophages in ovarian endometriosis was already reported in our previous preliminary study [30]. Until now RCAS1-positive macrophages were observed in the bone marrow, peripheral blood of patients with Hodgkin lymphoma, nasal polyps and in immune mediated liver disease [11,31,32]. Perhaps the alteration of macrophage-NK cells and macrophage-cytotoxic T lymphocyte interactions might help the cells survive in ectopic localization. In scar endometriomas no RCAS1-positive macrophages were observed, but this is the situation when the primary lesion develops during the immune tolerance against fetal antigens in pregnancy maintenance and this protection seems to facilitate the development of the endometriomas foci. Our results seem to confirm the clinical observations of scar endometriomas. Although the implantation of decidual cells might be induced iatrogenically by mechanical transplantation, endometriomas do not develop in all scars following cesarean sections and occur more frequently following elective cesarean sections performed during the maintenance of immune tolerance during pregnancy [33-35].

Implanting endometrial cells within the cesarean scar possesses both the ability to regulate the activity of immune response and a high resistance to immune mediated apoptosis similar to eutopic endometrial cells during the secretory cycle phase [6,29]. The recruitment of lymphocytes to decidua takes place during the whole course of pregnancy [36], pregnancy termination is related with the termination of their activity inhibition [37], decidual cells are extremely exposed to immune mediated apoptosis. The resistance to immune mediated apoptosis of the endometrial cells can be related to MT expression [38-41]. The increase of MT endometrial immunoreactivity during the implantation window seems to be secondary to the growing cytotoxic activity. A higher level of MT expression has been demonstrated in the eutopic endometrium simultaneously with the increasing number and activity of cytotoxic cells [10]. Endometriosis cells are characterized by the inability to transmit a "death" signal [42]. In the present study MT expression in ovarian endometriosis was at the lowest level when compared to the eutopic endometrium and scar endometriosis. However, it was at the comparable level in scar endometriosis and the control group. High MT expression in scar endometriomas was accompanied by the growing number of CD25- and CD69-positive immune cells, while low MT expression in ovarian endometriomas accompanied by a lower number of CD25- and CD69-positive immune cells coexisted with the presence of RCAS1-positive macrophages. Thus, macrophages probably play an additional role in the NK cells activity restrictions. Moreover, differences in MT expression between epithelial cells and stroma were identified, what seems to confirm the reports on differences between apoptosis levels in the glandular epithelium and stroma in ovarian endometrioid cyst [43,44].

The expression of RCAS1 and metallothionein by endometrial cells is important for the coexistence of activated immune cells together with endometrial cells during decidualization in secretory cycle phase. This expression may favor the persistence of endometrial cells in ectopic localization both in scar following cesarean section and in ovarian endometriosis.

## Abbreviations

Receptor associated cancer antigen presenting on SiSo cells (RCAS1); metallothionein (MT); class I human lymphocyte antigens (HLA-I); natural killer cells (NK); tumor necrosis factor (TNF); cytotoxic T lymphocytes (CTLs).
